# Phantom‐based comparative analysis of contrast‐enhanced mammography systems: Image quality and performance evaluation

**DOI:** 10.1002/acm2.70163

**Published:** 2025-07-14

**Authors:** Giulia Bruschi, Valerio Ricciardi, Paolo De Marco, Daniela Origgi

**Affiliations:** ^1^ School of Medical Physics University of Milan Milan Italy; ^2^ Medical Physics Unit IEO European Institute of Oncology IRCCS Milan Italy

**Keywords:** average glandular dose, CEM, image quality, quantification

## Abstract

**Purpose:**

Contrast‐enhanced mammography (CEM) is a technique that exploits the combination of two projections at different energies to produce an energy‐subtracted (ES) image to highlight the uptake of iodinated contrast medium in breast lesions. The aim of this study is to analyze and compare the performance of different CEM systems.

**Methods:**

Four mammography systems of different vendors (Fujifilm Amulet Innovality, Hologic 3Dimensions, IMS Giotto Class, GE Senographe Pristina) were compared employing a commercial dedicated phantom, equipped with a breast‐equivalent target slab with four different iodine concentrations (IC) inserts. Acquisition parameters and average glandular dose (AGD) were collected at different phantom thicknesses (30–80 mm). Contrast‐to‐noise ratio (CNR) and linearity of IC response with phantom thickness were evaluated.

**Results:**

AGDs (mGy) were in ranges 0.82–3.10, 0.90–6.25, 1.08–3.30, and 0.74–3.63 for Fujifilm, Hologic, GE, and IMS, respectively. High‐energy AGD accounted for up to 41.5%, 25.6%, 40.5%, and 23.8% of the total dose for Fujifilm, Hologic, IMS, and GE, respectively. CNR increased with IC and generally decreased with increasing phantom thickness. In recombined images, Hologic, Fujifilm, and GE showed good linearity of IC response with phantom thickness and overlapping trends (maximum error = 1%) for all thicknesses.

**Conclusion:**

Similarities and differences in image quality and dose were found depending on the different technical and image processing characteristics of the different vendors. Linearity of IC at various thicknesses might be further exploited in clinical scenarios to differentiate between suspicious breast lesions.

## 1 INTRODUCTION

Breast cancer is one of the most common types of tumor that affects women and, with 2.3 million estimated new cases in 2020, it has become the world's most diagnosed cancer.^1^ Therefore, breast imaging plays a pivotal role in early diagnosis to improve patients’ lives and reduce mortality.

Contrast‐enhanced mammography (CEM) is an emerging technique for the assessment of lesion enhancement in dense breasts using an intravenously injected iodinated contrast medium. It employs the acquisition of two consecutive projections at different x‐ray energies to produce an energy‐subtracted (ES) image that highlights the enhancing tissues through the uptake of the iodinated contrast medium. The projection at lower energy (LE image) is equivalent to a conventional FFDM, where the x‐ray absorption by the contrast medium is negligible, while the second image, at higher energy level (HE image), allows the maximum attenuation by iodine. The ES recombined image highlights the contrast‐enhanced regions.^2^ For these characteristics, CEM is able to combine morphological information from FFDM with functional information, similarly to breast MRI.

The high performance of CEM in detecting and diagnosing breast lesions is already well described in the literature: it has a sensitivity comparable to breast MRI (91.5%) with a lower false positive rate (10.5% for CEM vs. 19.8% for breast MRI), lower costs, less time for examination, and more comfort for patients.^3^

The characterization of CEM mammography systems and their performance evaluation are still under study and not yet completed for all commercial units. The aim of this study is to analyze and compare four mammography systems of different vendors, in terms of average glandular dose (AGD), contrast‐to‐noise ratio (CNR), and iodine quantification, employing a commercial phantom designed specifically for CEM quality assurance.

## 2 METHODS

Acquisitions have been performed on four mammography units of different vendors installed at our institution. All systems’ performances have been tested during routine quality control procedures, executed immediately before or in the days preceding the measurements.

### 2.1 Mammography units

The mammography units are the following:

- Fujifilm Amulet Innovality (Fujifilm, Minato, JAP)
- Hologic 3dimensions (Hologic Inc., Marlborough, Massachusetts, USA)
- IMS Giotto class (IMS Giotto, Sasso Marconi, ITA)
- GE Senographe Pristina (General Electrics Company, Boston, Massachusetts, USA)

Investigated systems differ in anode/filter combination, detector type (direct or indirect x‐rays conversion), pixel dimension and AEC modes; their main features are summarized in Table 1.

**TABLE 1:** Technical specifications of the mammographic units used in the study for CEM modality.

Model	Fujifilm Amulet Innovality	Hologic 3Dimensions	GE Senographe Pristina	IMS Giotto Class
Installed in	2018	2020	2018	2022
Anode	W	W	Mo, Rh	W
Filtration (thickness in mm)	Rh (0.05); Al (0.7); Cu (0.25)	Rh (0.05); Al (0.7); Ag (0.05); Cu (0.3)	Ag (0.03); Mo (0.03); Cu (0.25)	Ag (0.05), Cu (0.3)
Detector type	a‐Se	a‐Se	CsI	a‐Se
Detector size (mm^2^)	240 × 300	240 × 300	240 × 300	240 × 300
Pixel dimension (mm)	0.05	0.07	0.1	0.085
AEC mode	–	8 level of AEC compensation (‐3; …; +4)	STD	Standard, Contrast
AEC sensor	Manual, auto, i‐AEC	Manual, auto	Auto	Auto (area selection)
Software version	V9.3.6356.0002	1.10.0.421	3.4.51	Raffaello 4.14.5.0 ‐ IMSProc 4.14.2.0

### 2.2 Phantom

**FIGURE 1:**
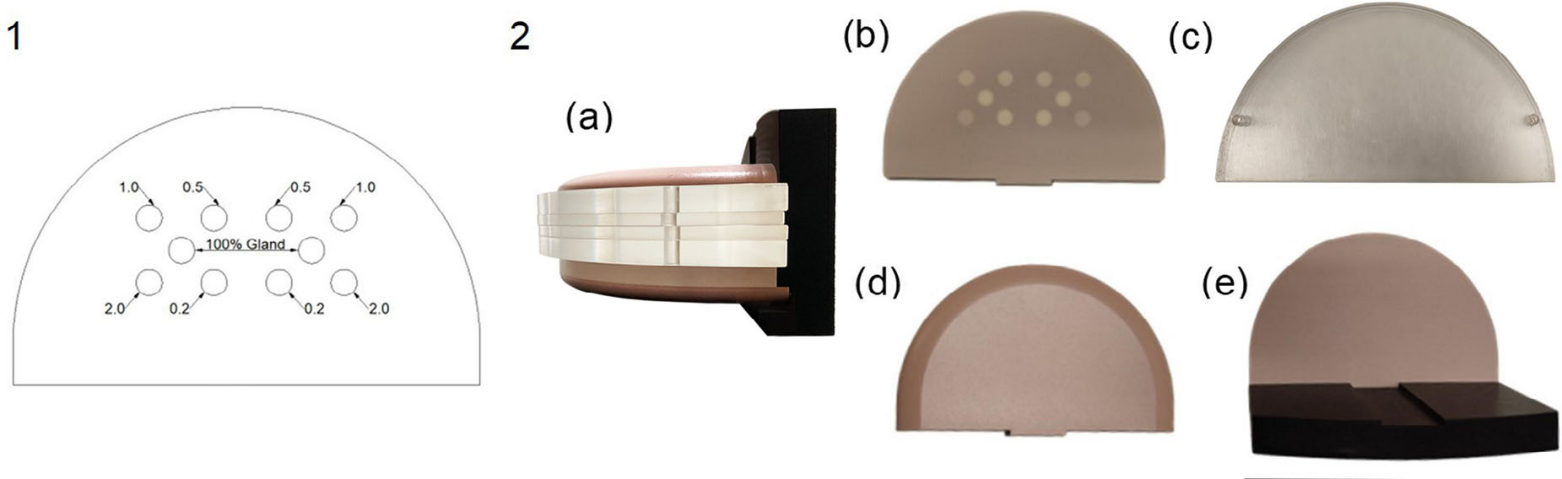
CIRS phantom used for measurements: (1) scheme of iodine inserts in the contrast slab (inserts ICs are reported in mg/cm^2^); (2) photo of the phantom: (a) whole phantom, (b) insert slab, (c) PMMA slabs, (d) top slab, and (e) bottom slab with position holder.

The phantom used in this study is the CIRS model 022 (Sun Nuclear, Melbourne, Florida, USA), specifically designed for CEM evaluations (Figure 1).

This model includes a position holder with magnetic fixation, two upper and lower 10 mm thick homogeneous slabs made of adipose‐mimicking tissue, designed to imitate the realistic shape of the breast, and a 10 mm thick central target slab with iodine inserts, creating a total thickness of 30 mm. There are five cylindrical inserts (diameter = 10 mm) in each half of the target slab, symmetrically positioned. The central insert is made of glandular equivalent material, while the others have ICs of 2.0, 1.0, 0.5, and 0.2 mg/cm^2^. PMMA slabs were added to study the systems response across phantom thicknesses ranging from 30 to 80 mm (30, 40, 45, 50, 60, 70, and 80 mm).

### 2.3 Acquisitions

The phantom was positioned on the detector plates of each system using the position holder to attach it to the center of the side corresponding to the patient's chest wall. Acquisitions were performed using automatic exposure control (AEC) for each system, as normally done during clinical practice, with a constant compression force of 50N; displayed thickness was within 1 mm of the nominal phantom thickness for each system.

In the GE system, the automatic AEC can utilize any region of the detector to select the exposure parameters during pre‐irradiation. The Hologic system automatic AEC utilizes two of seven possible regions within the central strip of the detector, based on breast size. Eight different AEC compensation steps can be used to modulate the mAs to increase CNR (+1 to +4) or decrease dose (‐1 to ‐3); the “0” step was used during the acquisitions. The automatic AEC of the IMS system uses a sensitive area at the center of the detector; the Standard mode, used in clinical practice, was employed for this study. The Fujifilm AEC system allows the user to choose between manual, automatic and i‐AEC modes. Auto AEC detects the highest attenuation region using multiple sensors, while i‐AEC identifies the mammary gland region based on the morphological characteristics recognized within the images. Fujifilm images were acquired using the auto AEC mode rather than the i‐AEC mode, which is normally used in clinical examinations but is not suitable for performance evaluation using phantoms with high contrast inserts.^4^

The anode/filter combination, HVL, kVp, mAs, and AGD were extracted from the DICOM files of the acquired images in order to compare the exposure parameters.

It is worth noting that all devices use Dance model for AGD estimation,^5^, ^6^, ^7^, ^8^ so no further conversion was needed to compare those values.

### 2.4 Image analysis

**FIGURE 2:**
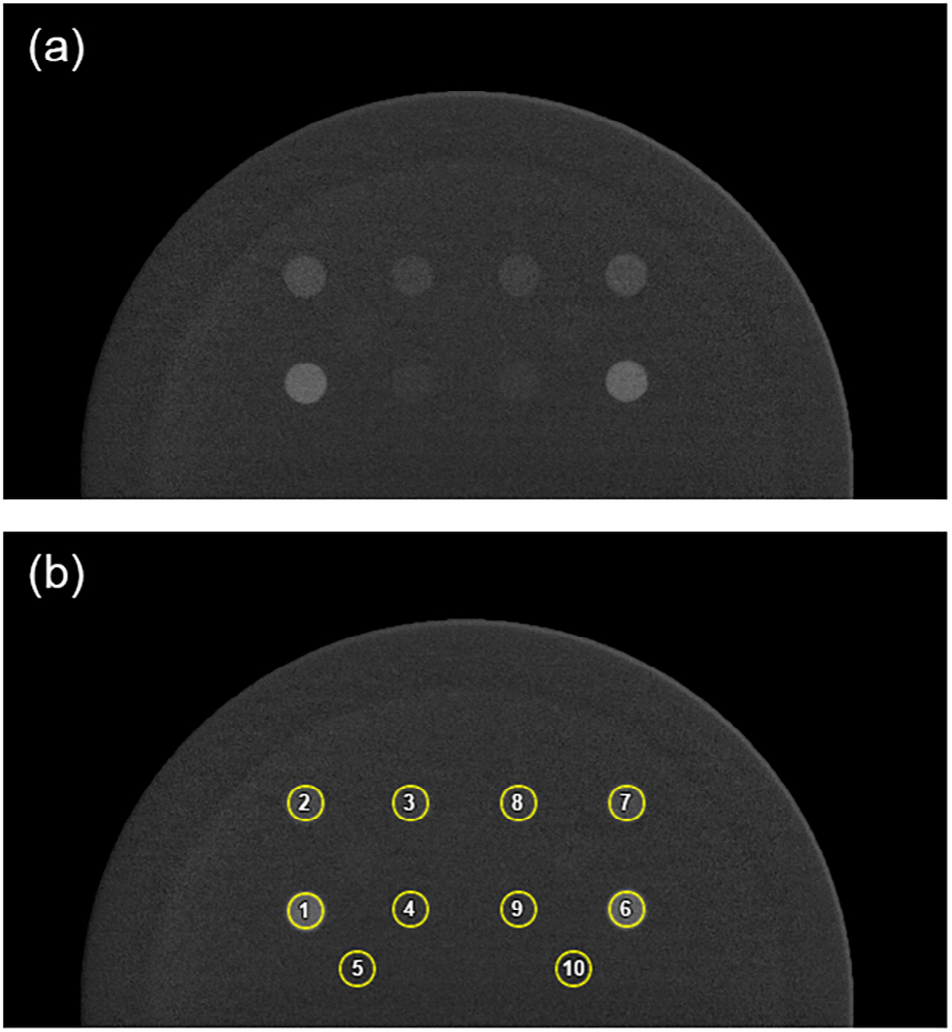
Example of ROIs placing used for the analysis: (a) ES image acquired with the GE system in the “30 mm” configuration, (b) the same image with the ROIs applied. ROIs from 1–4 and 6–9 are positioned on the iodine inserts (2, 1, 0.5, and 0.2 mg/cm^2^, respectively), while ROIs 5 and 10 on the background.

The analysis was performed on the ES images using the opensource software *ImageJ*.^9^ Circular ROIs (diameter = 8 mm) were positioned at the center of each iodinated insert and in the background (Figure 2). Mean pixel value (MPV) and its standard deviation (SD) were extracted from each ROI. No significant difference was observed between the values calculated for the corresponding ROIs on the left and right sides of the phantom, including the background, for all the systems. Therefore, the analysis was conducted using only the left‐side inserts (ROIs 1–5) for all images.

Contrast‐to‐noise ratio (CNR) was calculated for each insert and each thickness as

(1) $$\mathrm{CNR}\;=\frac{\mathrm{MP}V_{\mathrm{insert}\;}-\;\mathrm{MP}V_{\mathrm{background}}}{SD_{\mathrm{background}}}$$

A further analysis was performed to evaluate the relationship between IC and MPV as a function of thickness, using background ROI as null concentration.^10^

In addition, the average MPV values for a given concentration at different thicknesses were evaluated in order to understand if the systems could be described by a simple linear regression, regardless of phantom thickness.

## 3 RESULTS

**FIGURE 3:**
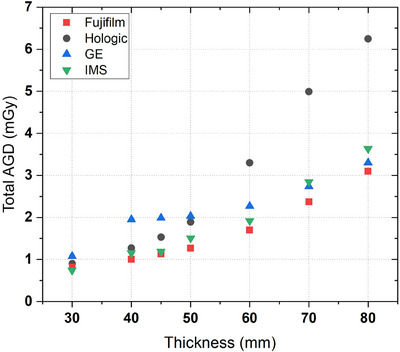
CEM total AGD for all the mammography devices, at different phantom thickness.

The exposure parameters used for LE and HE images at different phantom thickness are presented in Table 2, while the total AGD values are reported in Figure 3.

**TABLE 2:** Exposure parameters for LE and HE acquisitions, at thickness from 30 and 80 mm for all the mammography units (A/F = anode/filter).

	Fujifilm					Hologic					GE					IMS				
Thickness (mm)	A/F	kV	mAs	HVL (mm Al)	AGD (mGy)	A/F	kV	mAs	HVL (mm Al)	AGD (mGy)	A/F	kV	mAs	HVL (mm Al)	AGD (mGy)	A/F	kV	mAs	HVL (mm Al)	AGD (mGy)
LE	30	W/Rh	26	36	0.51	0.48	W/Rh	26	58	0.52	0.67	Mo/Mo	26	28	0.34	0.7	W/Al	33	15	0.58	0.58
	40	W/Rh	28	44	0.53	0.66	W/Rh	28	74	0.54	0.96	Rh/Ag	34	22	0.49	1.16	W/Al	34	23	0.60	0.88
	45	W/Rh	28	53	0.53	0.76	W/Rh	28	95	0.54	1.16	Rh/Ag	34	26	0.49	1.23	W/Al	34	26	0.60	0.94
	50	W/Rh	28	65	0.53	0.91	W/Ag	29	86	0.56	1.44	Rh/Ag	34	31	0.49	1.31	W/Al	34	33	0.60	1.16
	60	W/Rh	30	83	0.55	1.31	W/Ag	31	133	0.57	2.53	Rh/Ag	34	44	0.49	1.57	W/Al	34	48	0.60	1.55
	70	W/Rh	30	131	0.55	1.93	W/Ag	31	215	0.57	3.83	Rh/Ag	34	65	0.49	2.10	W/Al	35	70	0.61	2.36
	80	W/Rh	31	172	0.56	2.59	W/Ag	32	264	0.58	4.84	Rh/Ag	34	92	0.49	2.60	W/Al	35	96	0.61	3.04
HE	30	W/Cu	45	47	3.37	0.34	W/Cu	45	40	3.18	0.23	Mo/Cu	49	59	2.94	0.38	W/Cu	45	33	2.90	0.16
	40	W/Cu	45	50	3.37	0.35	W/Cu	45	56	3.18	0.31	Rh/Cu	49	117	2.87	0.79	W/Cu	45	42	2.90	0.28
	45	W/Cu	45	54	3.37	0.37	W/Cu	45	67	3.18	0.37	Rh/Cu	49	115	2.87	0.76	W/Cu	45	48	2.90	0.25
	50	W/Cu	45	52	3.37	0.36	W/Cu	49	51	3.46	0.45	Rh/Cu	49	113	2.87	0.72	W/Cu	46	53	2.96	0.35
	60	W/Cu	45	59	3.37	0.39	W/Cu	49	90	3.46	0.77	Rh/Cu	49	116	2.87	0.70	W/Cu	46	63	2.96	0.37
	70	W/Cu	45	69	3.37	0.44	W/Cu	49	140	3.46	1.16	Rh/Cu	49	114	2.87	0.64	W/Cu	46	73	2.96	0.48
	80	W/Cu	45	84	3.37	0.51	W/Cu	49	176	3.46	1.41	Rh/Cu	49	134	2.87	0.70	W/Cu	47	83	3.06	0.59

Systems employed different anode/filter combinations for LE acquisitions: W/Rh for Fujifilm, with increasing kVp and mAs with thickness; W/Rh and W/Ag (above 45 mm) for Hologic, with increasing kVp and mAs values with thickness, with the latter higher than all the other systems; W/Al for IMS, with both kVp and mAs increasing with thickness while GE used Mo/Mo and Rh/Ag (above 30 mm) with fixed two‐step kVp.

Fujifilm, IMS, and Hologic used W/Cu combination for HE acquisitions: while Fujifilm kept the kVp fixed with increasing mAs values, IMS used increasing values for both kVp and mAs and Hologic used a two‐step kVp choice, with mAs values increasing with thickness with higher values compared to the other systems at the largest thickness (above 60 mm). GE used Mo/Cu and Rh/Cu (above 30 mm), with fixed kVp and mAs values that almost doubled above 30 mm and remained stable for higher thickness.

AGD values for HE projections represented 22.6%–25.6% of total AGD for the Hologic system, 21.2%–40.5% for GE, 16.2%–23.8% for IMS, and 16.5%–41.5% for Fujifilm.

**FIGURE 4:**
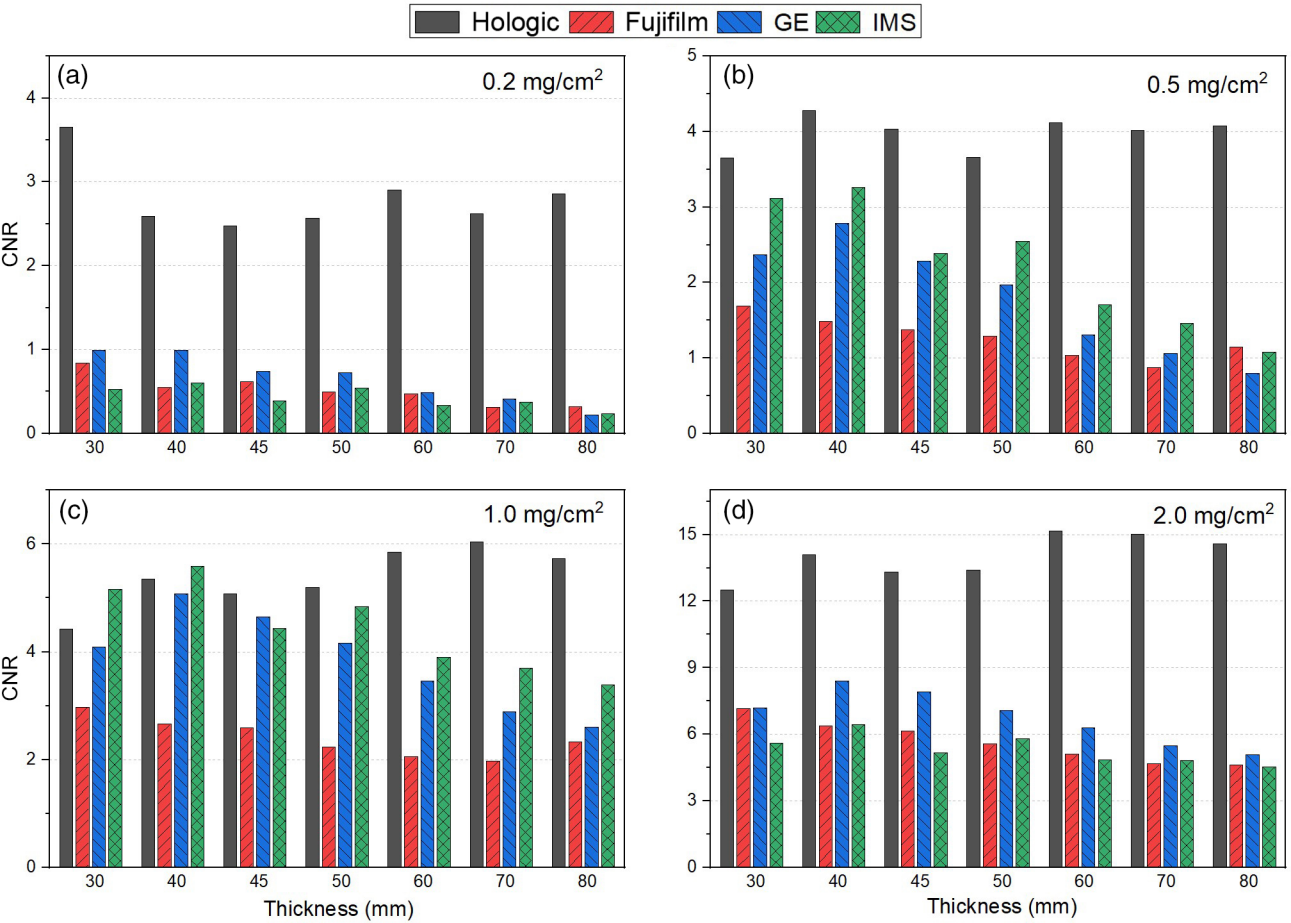
CNR for all the mammography devices, at different phantom thickness and IC.

CNR as a function of thickness is presented in Figure 4. Hologic demonstrated notably greater CNR, roughly twice the other three vendors, across nearly all concentrations and thickness, with the exception of 30 and 40 mm acquisitions at 1 mg/cm^2^. Fujifilm, GE and IMS exhibited similar CNRs across thickness for the lowest and highest ICs. However, Fujifilm tended to underperform relative to GE and IMS for CNRs at intermediate ICs, though this difference diminished with increasing thickness.

**FIGURE 5:**
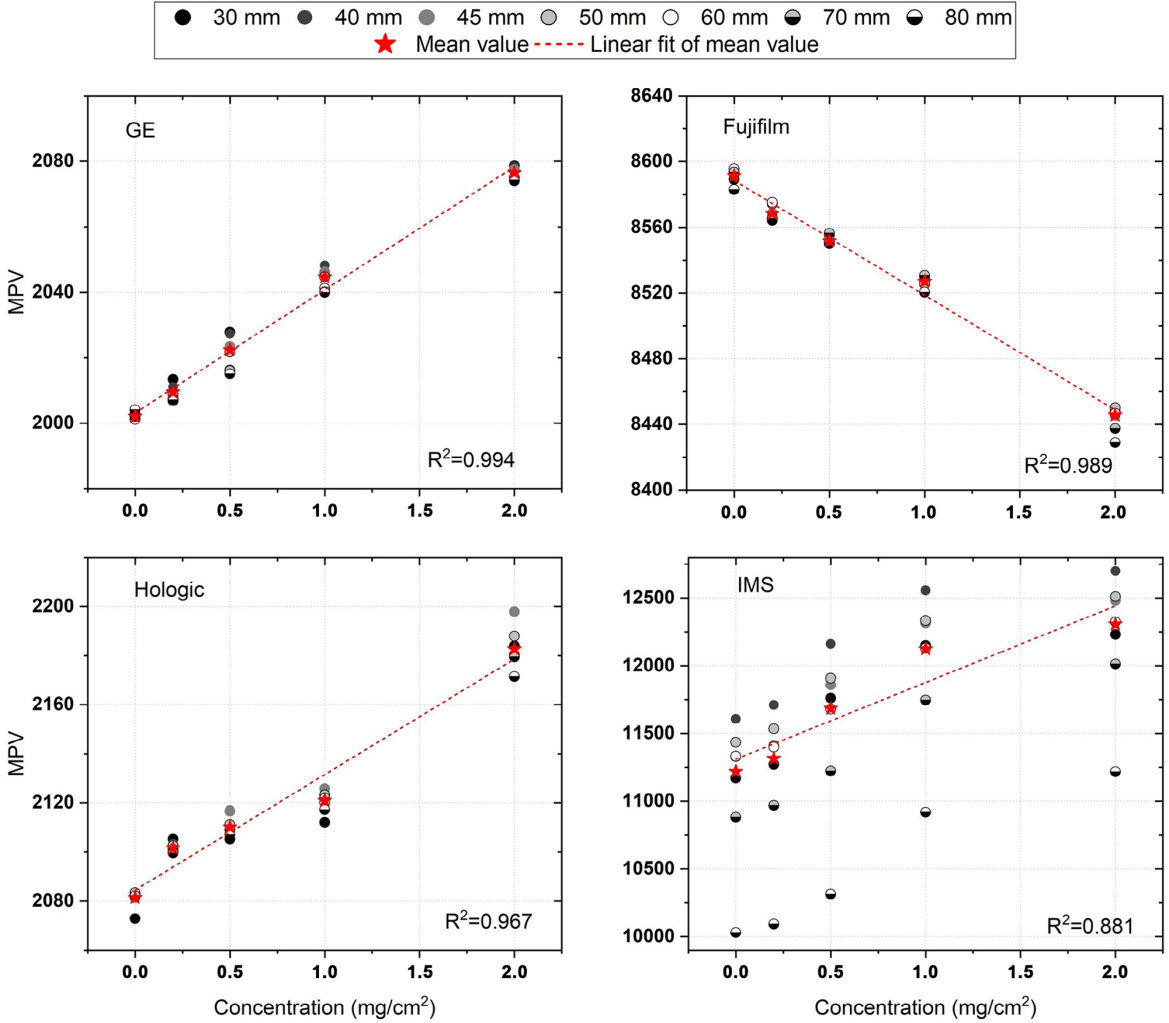
MPVs for the different ICs at varying phantom thickness for all the mammography devices; linear fits for the average values of MPVs at different thickness together with corresponding *R* ^2^ scores are also reported.

The results of the analysis of the relationship between IC and MPV are reported in Figure 5, with the linear fit for the MPVs averaged over the different thicknesses.

Both Fujifilm and GE showed a good linear relationship between IC and MPV for all thicknesses (*R* ^2^ = 0.99); the Hologic unit showed a less linear relationship between IC and MPV (*R* ^2^ = 0.97) while IMS showed a different behavior compared to the others with MPV varying greatly with thickness. Notably, linear regression performed on the average MPV values over all thicknesses for each IC resulted in maximum errors of 0.2%, 1%, and 0.4% for Fujifilm, Hologic, and GE, respectively.

## 4 DISCUSSION

Four different CEM systems were compared in terms of AGD, CNR, and response between MPV and IC.

### 4.1 Exposure parameters

Hologic use of different filters in LE acquisitions depending on the phantom thickness was also reported by the technical report of the NCCPM on this unit.^11^ The higher AGD values with increasing phantom thickness (60 mm or greater) were reported also by Ghetti et al.^12^ and can be justified by the AEC selected parameters.

For the GE system, the AEC‐selected parameters resulted in a sudden increase of AGD above 30 mm and higher values, compared to other systems, for lower thicknesses (below 60 mm). This behavior was confirmed by the NCCPM technical report^13^ and periodical quality control performed on that unit.

For both IMS and Fujifilm, the selected parameters led to an expected AGD increase with thickness, although the values for Fujifilm remained the lowest compared to the other systems across all thicknesses except for 30 mm.

### 4.2 CNR

The Hologic system showed a different behavior compared to the other systems. For thicknesses from 50 mm and above, Hologic used a different filter for LE acquisitions and produced higher AGD values (Figure 3): it is possible that the system tries to preserve CNR at higher thicknesses at the cost of increasing patient exposure (Figure 4). For GE, the CNR values decreased monotonically with thickness, except for 30 mm where a lower CNR was observed (Figure 4); this could be explained considering the difference in terms of anode/filter combination and exposure parameter selected above this thickness. The CNR trend observed for GE and Hologic is consistent with those found by Gennaro et al.^14^ However, absolute values are not directly comparable because Gennaro et al. utilized a PMMA phantom with a 15 × 15 mm^2^ aluminium contrast object with 0.2 mm thickness.

The reported results illustrate the technical performance differences arising from manufacturers’ choices and technologies. However, it is important to note that the metrics used serve as surrogate of image quality, and clinical performance of the investigated devices cannot be deduced from this study.

### 4.3 Linearity

The analysis performed on the relationship between IC and MPV on ES images demonstrated that three of the four systems (Fujifilm, GE, and Hologic) show MPVs that are independent of phantom thickness for the different ICs (Figure 5).

In particular, the Fujifilm unit, which is the only one that uses an inverted look‐up table (LUT) with MPVs decreasing with the increasing ICs, presented reproducible linear fit parameters for different phantom thickness and *R* ^2^ scores in the range [0.974–0.995]. In addition, the linear fit on mean values provides a linear function of correlation between MPV and IC (*R* ^2^ = 0.989), with percentage differences lower than 0.2% between the mean value regression score and the measured ones, resulting in a good approximation across all thicknesses and concentrations.

Similarly, the GE unit exhibited comparable linear fit parameters across all thicknesses and a good linear correlation highlighted by high *R* ^2^ values (range [0.985–0.995]). The linear fit based on mean values resulted in an *R* ^2^ = 0.994 and a maximum percentage discrepancy lower than 0.4%.

The Hologic system showed similar results for different thicknesses, but *R* ^2^ values of their regressions were lower (range [0.914–0.989]). Additionally, for the linear regression on mean values, a lower *R* ^2^ value was found (*R* ^2^ = 0.967) indicating a less strong linear correlation between MPVs and ICs; despite this, the discrepancy between the obtained fit and the thickness‐dependent datasets was lower than 1%.

The IMS system showed different behavior, with measured MPVs varying greatly with phantom thickness. Moreover, in these cases, *R* ^2^ values were lower than 0.95 for all the thicknesses (range [0.801–0.931]), and so, their mean values were not considered as an appropriate approximation of the data. This behavior could be attributed to the IMS proprietary recombination algorithm of LE and HE images.

Linearity of MPV was also studied by Ghetti et al.^12^ and Cockmartin et al.^15^ using the complete CIRS phantom model 022 with 55 mm of thickness. Although their results are not directly comparable with regression coefficients found in this work, due to the different types of analysis and the use of a fixed phantom thickness, the linearity values reported can be discussed. Fujifilm and GE demonstrated linear response in both studies resulting in values consistent with the findings of this work. Cockmartin reported Hologic as linear, with *R* ^2^ values in the range [0.97–0.98], while Ghetti found an *R* ^2^ in the range [0.94–0.95]. Furthermore, the results on IMS differ from those found by Ghetti (*R* ^2^ in the range [0.98–0.99]). When comparing results on processed images with other studies, however, it is important to consider the software versions employed, as variations in recombination algorithms across software versions can affect the characteristics of CEM recombined images.

Such simple relationships between MPVs and concentrations across the studied thicknesses suggest the potential for clinical application. In fact, by using MPVs from subtracted images it is feasible to quantify the contrast medium concentration,^10^ which could aid in differentiating between malignant and benign lesions.^16^ This quantification would be much easier for three systems (Fujifilm, GE, Hologic), given the small magnitude of the error (less than 1% for all systems) when considering a single linear relationship for all thicknesses. For IMS, however, it would be necessary to modify the processing algorithm to produce a linear pixel value response with IC at least for each thickness before quantification.

In conclusion, in this work, a technical comparison of four different CEM mammography systems was carried out in terms of dose, CNR, and system response for IC.

The systems investigated differ in anode/filter combination, detector type, and AEC modes; such differences are reflected in AGD and CNR results.

Since recombined images are used, post‐processing has a strong impact on systems response; although the phantom results presented here may not be directly applicable to clinical practice, useful indications and quantitative parameters can be obtained from such images.

The linear relationship between MPV of recombined images and ICs at different thicknesses would allow a direct iodine quantification that could be exploited in clinical images. Future clinical studies are expected to investigate the feasibility of such an approach.

## AUTHOR CONTRIBUTIONS

Giulia Bruschi is responsible of acquisition, analysis and interpretation of the data, and writing the manuscript. Valerio Ricciardi is responsible of acquisition, analysis and interpretation of the data, and editing the manuscript. Paolo De Marco and Daniela Origgi are responsible of interpretation of data and editing the manuscript.

## CONFLICT OF INTEREST STATEMENT

The authors declare no conflicts of interest.

## Supporting information




Supporting Information

## Data Availability

The data that support the findings of this study are available from the corresponding author upon reasonable request.

## References

[1] World Health Organization . Breast cancer information. https://www.who.int/news‐room/fact‐sheets/detail/breast‐cancer; 2023. Accessed February 9, 2024.

[2] Lobbes M , Jochelson MS , editors. Contrast‐Enhanced Mammography. 1st ed. Springer International Publishing; 2019. doi:10.1007/978-3-030-11063-5

[3] Xing D , Lv Y , Sun B , et al. Diagnostic value of contrast‐enhanced spectral mammography in comparison to magnetic resonance imaging in breast lesions. J Comput Assist Tomogr 2019; 43(2):245‐251. doi:10.1097/RCT.0000000000000832 30531546 PMC6426358

[4] Oda Y , Ito T , Sato K , et al. Development of digital mammography system “AMULET Innovality” for examining breast cancer [white paper]. Fujifilm Res Dev. 2014; 59:7‐9.

[5] Dance DR . Montecarlo calculation of conversion factor for the estimation of mean glandular breast dose. Phys Med Biol. 1990; 35(9):1211‐1219. doi:10.1088/0031-9155/35/9/002 2236205

[6] Dance DR , Skinner CL , Young KC , et al. Additional factors for the estimation of mean glandular breast dose using the UK mammography dosimetry protocol. Phys Med Biol. 2000; 45(11):3225‐3240. doi:10.1088/0031-9155/45/11/308 11098900

[7] Dance DR , Young KC , van Engen RE . Further factors for the estimation of mean glandular dose using the United Kingdom, European and IAEA breast dosimetry protocols. Phys Med Biol. 2009; 54(14):4361‐4372. doi:10.1088/0031-9155/54/14/002 19550001

[8] Dance DR , Young KC . Estimation of mean glandular dose for contrast enhanced digital mammography: factors for use with the UK, European and IAEA breast dosimetry protocols. Phys Med Biol. 2014; 59(9):2127‐2137. doi:10.1088/0031-9155/59/9/2127 24699200

[9] ImageJ RasbandWS . U. S. National Institutes of Health, https://imagej.net/ij/index.html. 2018. Accessed February 9, 2024.

[10] Lobbes MBI , Mulder HKP , Rousch M , et al. Quantification of enhancement in contrast‐enhanced spectral mammography using a custom‐made quantifier tool (I‐STRIP): a proof‐of‐concept study. Eur J Radiol. 2018; 106:114‐121. doi:10.1016/j.ejrad.2018.07.021 30150032

[11] Kelly M , Rai M , Mackenzie A . NCCPM Technical report 2003: technical evaluation of contrast enhanced mammography functions using Hologic I‐View software, https://medphys.royalsurrey.nhs.uk/nccpm/files/other/Tech_Eval_CESM_Hologic3Dimensions_Final.pdf; 2020. Accessed February 9, 2024.

[12] Ghetti C , Ortenzia O , Pagan L , et al. Physical and dosimetric characterisation of different contrast‐enhanced digital mammographic systems: a multicentric study. Phys Med 2024; 120:103334. doi:10.1016/j.ejmp.2024.103334 38520889

[13] Kelly M , Tyler N , Mackenzie A . NCCPM Technical report 2004: technical evaluation of SenoBright HD contrast enhanced mammography functions of Senographe GE Pristina system. https://medphys.royalsurrey.nhs.uk/nccpm/files/other/Tech_Eval_CESMGE_Pristina_NCCPMformatFinalV2.pdf; 2020. Accessed February 9, 2024.

[14] Gennaro G , Del Genio S , Manco G , Caumo F . Phantom‐based analysis of variations in automatic exposure control across three mammography systems: implications for radiation dose and image quality in mammography, DBT, and CEM. Eur Radiol Exp. 2024; 8(49). doi:10.1186/s41747-024-00447-z PMC11018565 38622388

[15] Cockmartin L , Bosmans H , Marshall NW . Investigation of test methods for QC in dual‐energy based contrast‐enhanced digital mammography systems: I. Iodine signal testing. Phys Med Biol. 2023; 68(21):215017. doi:10.1088/1361-6560/ad027d 37820689

[16] Deng CY , Juan YH , Cheung YC , et al. Quantitative analysis of enhanced malignant and benign lesions on contrast‐enhanced spectral mammography. Br J Radiol. 2018; 91(86):20170605. doi:10.1259/bjr.20170605 29451413 PMC6223273

