# Supplementary material for: Phantom‐based comparative analysis of contrast‐enhanced mammography systems: Image quality and performance evaluation
Source: J Appl Clin Med Phys. 2025 Jul 14;26(7):e70163. doi: 10.1002/acm2.70163 (PMC12257344; doi:10.1002/acm2.70163)
Supplement: Supplementary file 1 — Supporting Information [file ACM2-26-e70163-s001.docx]

**Title:**

Phantom-based comparative analysis of contrast-enhanced mammography systems: image quality and performance evaluation

**Authors:**

- Giulia Bruschi, M.S.

School of Medical Physics

University of Milan

Via Celoria 16

20133 Milan, Italy

Medical Physics Unit

IEO European Institute of Oncology IRCCS

Via Ripamonti 435

20141 Milan, Italy

[giulia.bruschi@unimi.it](mailto:giulia.bruschi@unimi.it)

- Valerio Ricciardi, Ph.D. (corresponding author)

School of Medical Physics

University of Milan

Via Celoria 16

20133 Milan, Italy

Medical Physics Unit

IEO European Institute of Oncology IRCCS

Via Ripamonti 435

20141 Milan, Italy

phone: 0294372165

[valerio.ricciardi@ieo.it](mailto:valerio.ricciardi@ieo.it)

- Paolo De Marco, M.P.E.

Medical Physics Unit

IEO European Institute of Oncology IRCCS

Via Ripamonti 435

20141 Milan, Italy

[paolo.demarco@ieo.it](mailto:paolo.demarco@ieo.it)

- Daniela Origgi, M.P.E.

Medical Physics Unit

IEO European Institute of Oncology IRCCS

Via Ripamonti 435

20141 Milan, Italy

[daniela.origgi@ieo.it](mailto:daniela.origgi@ieo.it)

**Running title**

Phantom-based comparative analysis of CEM systems

**Author contribution statement**

Giulia Bruschi is responsible of acquisition, analysis and interpretation of the data, and writing the manuscript. Valerio Ricciardi is responsible of acquisition, analysis and interpretation of the data, and editing the manuscript. Paolo De Marco and Daniela Origgi are responsible of interpretation of data and editing the manuscript.
